# Effect of initial drainage method on retrograde intrarenal surgery outcomes in acute calcular pyelonephritis: a prospective comparative study

**DOI:** 10.1007/s00240-025-01933-8

**Published:** 2026-01-27

**Authors:** Rabie M. Ibrahim, Amr M. Lotfy, Ossama Mahmoud, Mohamed Abd Elrady, Mahmoud Abdallah

**Affiliations:** Urology department, Faculty of medicine, Beni-Suef University, Beni-Suef, Egypt

**Keywords:** Percutaneous nephrostomy, Double-J stent, Obstructive pyelonephritis, Retrograde intrarenal surgery, Urolithiasis

## Abstract

This prospective study compared percutaneous nephrostomy (PCN) and double-J (DJ) stent drainage in 200 patients presenting with acute calcular pyelonephritis due to a single upper urinary tract stone ≤ 2 cm. Patients were randomized into two equal groups; PCN was performed under local anesthesia with ultrasound guidance, while DJ stenting was carried out under spinal anesthesia, and definitive retrograde intrarenal surgery (RIRS) was scheduled two weeks after drainage. Clinical recovery, operative parameters, postoperative complications, stone-free rates, and quality of life were assessed. Baseline demographic data and stone characteristics were comparable between groups. PCN achieved significantly faster normalization of temperature (3.5 ± 0.7 vs. 6 ± 1.4 h, *p* < 0.0001), earlier leukocyte count recovery (2.5 ± 0.6 vs. 3.5 ± 0.6 days, *p* < 0.0001), and shorter hospitalization (2.5 ± 0.6 vs. 3.5 ± 0.6 days, *p* < 0.0001). RIRS following PCN showed shorter operative time (55.1 ± 5.9 vs. 71.5 ± 3.2 min, *p* < 0.0001) and a lower rate of postoperative fever (5% vs. 20%, *p* = 0.002), while stone-free rates were similar (96% vs. 93%, *p* = 0.072). These findings suggest that PCN provides faster sepsis resolution and more favorable perioperative outcomes compared with DJ stenting, although both approaches allow successful subsequent RIRS.

## Introduction

Calcular obstructive pyelonephritis arises when infection develops in the setting of upper urinary tract obstruction caused by renal or proximal ureteric calculi [1, 2]. Patients usually present with flank pain, nausea, vomiting, and systemic manifestations such as fever, chills, and malaise. Without timely intervention, this condition can progress to severe complications including sepsis, renal abscess formation, or irreversible renal impairment [2, 3]. Urosepsis represents a significant clinical burden, accounting for nearly one quarter of all sepsis cases in adults [3, 4].

Prompt initiation of broad-spectrum antimicrobial therapy together with urgent urinary decompression is critical to reduce morbidity and prevent life-threatening outcomes [2, 4]. Current guidelines recommend temporary drainage using either a double-J (DJ) ureteral stent or percutaneous nephrostomy (PCN) before proceeding to definitive stone removal. Although ureteroscopy is associated with favorable stone-free rates, its role in infected systems remains controversial due to concerns about precipitating or worsening sepsis [5]. Some investigators have reported that PCN offers advantages over DJ in terms of patient comfort and effectiveness of drainage [6, 7], whereas other studies did not find significant differences between the two approaches [8, 9].

Despite numerous studies addressing PCN and DJ in obstructive pyelonephritis, most reports evaluating their influence on subsequent retrograde intrarenal surgery (RIRS) were retrospective and limited in scope [10]. In daily practice, the presence of a DJ stent often guides urologists to perform RIRS, while the presence of a PCN tract may encourage selection of percutaneous nephrolithotomy (PCNL). However, current evidence remains inconclusive regarding whether PCN reduces postoperative infectious complications compared with DJ drainage in patients later undergoing RIRS [7]. The present prospective study was therefore designed to compare PCN and DJ stent drainage in the setting of acute calcular pyelonephritis and to assess their impact on subsequent RIRS outcomes.

## Methodology

### Study design and settings

The study was a prospective randomized comparative study which included 200 patients whom attended the urology department, Beni-Suef University Hospital. Informed consents were signed from the participants after ethical approval from Beni Suef University Ethical committee number (FMBSUREC/01092024/Sorour).

### Participants

All patients developed urosepsis due to single proximal ureteral stones or kidney stones with a diameter of ≤ 2 cm were included. Patients with anatomical variations such as horseshoe kidney or pelvic kidney, and conditions like morbid obesity and bleeding disorders were excluded.

### Sample size

The sample size was calculated using G*power 3.1 based on the outcome analysis. Based on previous studies [11], the proportion of complication was 30.6% in PCN group and 51.8% for DJ stent group (*p* = 0.028). Calculated sample size for the current study is 200 (100 patients in each group), assuming a significant level (a) of 0.05, a power of 85%, 10% drop out, and ratio of sample sized in group 1: group 2 of 1:1 [12].

### Randomization

**Fig. 1:**
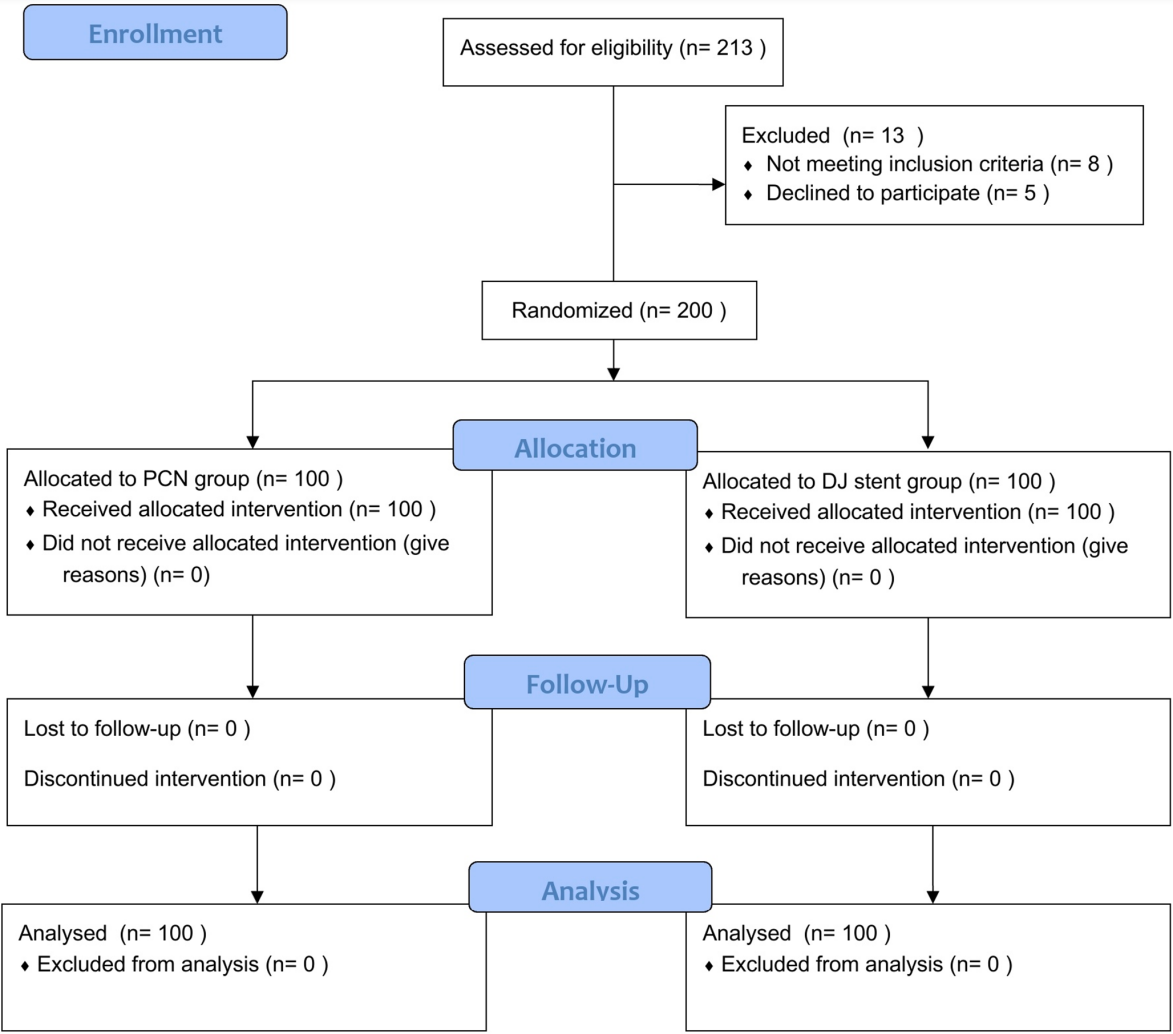
CONSORT flow diagram

Patients were randomly assigned to one of the two groups. The randomization was done using computer generated with 1:1 ratio. Treatment assignments were concealed using sequentially numbered, opaque, sealed envelopes, which were opened only after written consent and baseline assessments were completed. Figure 1 shows the flow diagram of the recruitment process.

### Study procedures

All patients were evaluated using non-contrast computed tomography, urinalysis, complete blood count, and urine culture to confirm the presence of a renal or proximal ureteral stone associated with urosepsis. The diagnosis of sepsis was based on established criteria, including abnormal body temperature (> 38 °C or < 36 °C), leukocytosis or leukopenia (> 11 or < 4 × 10⁹/L), and a quick Sequential Organ Failure Assessment (qSOFA) score of ≥ 2. The qSOFA score consisted of hypotension (systolic blood pressure ≤ 100 mmHg), tachypnea (respiratory rate ≥ 22 breaths/min), and altered mentation (Glasgow Coma Score < 15), each allocated one point to yield a total score between 0 and 3 [13]. Initial management involved intravenous fluid resuscitation and empirical use of broad-spectrum antibiotics, specifically third-generation cephalosporins, immediately upon diagnosis of urosepsis. This was subsequently adjusted to culture-directed therapy once the urine culture and sensitivity results became available. After emergency decompression and clinical stabilization, patients were scheduled for elective RIRS following a two-week interval.

### Drainage methods

**in PCN group**, an 8 or10F single step nephrostomy tube was inserted under ultrasound guidance after injection of subcutaneous 1% lidocaine anesthesia at the puncture site.

**In the DJ stent group**, A 6Fr DJ stent was advanced over a Zebra guidewire under fluoroscopy guidance under spinal anesthesia using ureteroscopic guidance (Richard Wolf semi rigid ureteroscope 8f).

### RIRS technique

Elective RIRS was performed once infection resolution was confirmed by normalization of leukocyte counts and sterile urine cultures. Procedures were carried out under general anesthesia. In **all** patients with DJ stents, an 11/13 Fr ureteral access sheath was introduced over a guidewire to allow passage of a 9.5 Fr digital flexible ureteroscope (Boston Scientific LithoVue). Stone fragmentation was achieved using a 100 W Holmium: YAG laser with a 200 μm fiber, applying dusting settings (0.5 J, 20 Hz), and manual irrigation with a 60 mL syringe. In the PCN group, the nephrostomy tube was left in situ during the procedure to help control intrapelvic pressure, and no access sheath was used. The nephrostomy tube was removed 24 h postoperatively. At the end of the procedure, a ureteric catheter or a 6 Fr DJ stent was inserted.

To ensure consistency and minimize confounding variables, a 9.5 Fr digital flexible ureteroscope (Boston Scientific LithoVue) was utilized for all Retrograde Intrarenal Surgery procedures, regardless of the initial drainage method (PCN or DJ stent) or the stone location (renal or ureteral). No semi-rigid ureteroscopes were used in this study.”

### Data collection

Preoperative assessment included demographic data, stone characteristics (size, location at renal pelvis/proximal ureter/PUJ), hydronephrosis severity according to the society of fetal urology [14], and comorbidities. Post-drainage evaluation documented time to fever resolution, leukocyte count normalization (The starting point for calculating the time to fever resolution and time to TLC normalization was the completion of the drainage procedure PCN insertion or DJ stenting), hospital stay duration, procedural success rates, complications, operative time, type of anesthesia and fluoroscopy exposure. RIRS outcomes encompassed operative time, stone-free rates (Non contrast CT-confirmed at 4 weeks), postoperative fever incidence (> 38 °C), catheter duration, access sheath utilization, postoperative hospitalization and quality of life (QoL). QoL was evaluated for all patients using the overall QoL and general health domains of the WHO quality of life scale by asking the patients how they rated their quality of life and how satisfied they were with their health, and each question had a score of 1 (very dissatisfied), 2 (dissatisfied), 3 (neither satisfied nor dissatisfied), 4 (satisfied), and 5 (very satisfied) [15].

### Statistical analysis

Data analysis utilized SPSS v23.0 (IBM) with appropriate tests for variable types: independent t-tests for normally distributed continuous data, Mann-Whitney U tests for non-parametric equivalents, and chi-square tests for categorical variables. Descriptive statistics included means, standard deviations, medians, ranges, and percentages. Statistical significance was set at *p* < 0.05 for all comparisons between the PCN and DJ stent groups.

## Results

One hundred patients undergoing PCN and another one hundred patients received DJ stent placement. The mean age, stone site and stone sizes, gender distribution, Hydronephrosis grade, comorbidities were comparable with no significant difference between the two groups. At admission, both groups presented with comparable inflammatory and clinical status. Mean body temperature was 39.3 ± 0.7 °C in the PCN group versus 39.1 ± 0.9 °C in the DJ group (*p* = 0.21), and initial TLC was 18.7 ± 4.3 versus 18.1 ± 4.1 × 10⁹/L, respectively (*p* = 0.32). The proportion of patients with qSOFA score ≥ 2 was similar between groups (40% PCN vs. 36% DJ, *p* = 0.54), indicating comparable severity of systemic inflammatory response at baseline. PCN achieved significantly faster TLC normalization compared with DJ drainage (2.5 ± 0.6 vs. 3.5 ± 0.6 days, *p* < 0.0001). However, the magnitude of TLC reduction from baseline to normalization was comparable between groups (16.2 ± 4.2 vs. 15.6 ± 4.0 × 10⁹/L, *p* = 0.28), suggesting that both drainage methods achieved similar absolute reduction in leukocytosis, although PCN did so more rapidly. Similarly, PCN achieved faster normalization of body temperature (3.5 ± 0.7 vs. 6 ± 1.4 h, *p* < 0.0001), reflecting superior and expedited control of the acute inflammatory response to sepsis (Table 1).

**Table 1:** Demographics and pre-operative of the participants

Variables		Total	PCN (*n* = 100)	DJ (*n* = 100)	*P* values
Age in years, mean (SD)		48.56 (14.66)	47.63 (15.46)	49.49 (13.83)	0.37
Gender, n (%)	Male	118 (59)	56 (56)	62 (62)	0.47
	Female	82 (41)	44 (44)	38 (38)	
Stone size in mm, mean (SD)		15.03 (3.21)	14.97 (3.25)	15.10 (3.18)	0.77
Stone site	PUJ	92 (46)	50 (50)	42 (42)	0.52
	Pelvic	24 (12)	11 (11)	13 (13)	
	Upper ureter	84 (42)	39 (39)	45 (45)	
Degree of hydronephrosis, n (%)	G1	17 (8.5)	7 (7)	10 (10)	0.84
	G2	133 (66.5)	69 (69)	64 (64)	
	G3	39 (19.5)	19 (19)	20 (20)	
	G4	11 (5.5)	5 (5)	6 (6)	
Comorbidities, n (%)	Non	29 (14.5)	14 (14)	15 (15)	0.64
	HTN	51 (25.5)	28 (28)	23 (23)	
	DM	106 (53)	53 (53)	53 (53)	
	Cardiac	14 (7)	5 (5)	9 (9)	
**Admission clinical status**					
**Temperature (°C)**,** mean (SD)**		**39.2 (0.8)**	**39.3 (0.7)**	**39.1 (0.9)**	**0.21**
**Initial TLC (×10⁹/L)**,** mean (SD)**		**18.4 (4.2)**	**18.7 (4.3)**	**18.1 (4.1)**	**0.32**
**qSOFA score ≥ 2**,** n (%)**		**76 (38)**	**40 (40)**	**36 (36)**	**0.54**
**TLC reduction (×10⁹/L)**,** mean (SD)**		**15.9 (4.1)**	**16.2 (4.2)**	**15.6 (4.0)**	**0.28**
**Time to TLC normalization (days)**,** mean (SD)**		**3.0 (0.7)**	**2.5 (0.6)**	**3.5 (0.6)**	< 0.0001*

The PCN group demonstrated superior early outcomes, with faster temperature normalization, total leukocyte count recovery, and shorter hospital stays *p* < 0.0001. PCN procedures were also more time-efficient regarding total operative time (16 ± 1.7 vs. 33.1 ± 5.2 min; *p* < 0.0001), with comparable complication rates (all are Grade 1–2) and similar initial success rates regarding to the procedure itself *p* = 0.72 and *p* = 0.64 respectively). Table 2

Additionally, during subsequent RIRS, the PCN group had shorter operative time; hospital stay, and catheter time *p* < 0.0001 respectively. Access sheath use was exclusively required in DJ group *p* < 0.0001). Postoperative fever incidence was significantly lower in PCN *p* = 0.002. While no significant difference ln stone-free rates (*p* = 0.072). QoL scores were comparable between groups *p* = 0.75. Table 2

**Table 2:** After procedure and RIRS variables

Variables		PCN	DJ	*P* value
**After procedure variables**				
Time to normalized body temperature in hours, mean (SD)		3.5 (0.7)	6 (1.4)	< 0.0001*
Time to normalize TLC in days, mean (SD)		2.5 (0.6)	3.5 (0.6)	< 0.0001*
Hospital stays in days, mean (SD)		2.5 (0.6)	3.5 (0.6)	< 0.0001*
Operative time in min, mean (SD)		16 (1.7)	33.1 (5.2)	< 0.0001*
Complications, n (%)	Grade 1	90 (90)	86 (86)	0.72
	Grade 2	10 (10)	14 (14)	
Success rate, n (%)	Yes	100 (100)	90 (90)	0.64
	No	0 (0)	10 (10)	
**After and during RIRS variables**				
Operative time in min, mean (SD)		55.15 (5.88)	71.5 (3.21)	< 0.0001*
Hospital stays, mean (SD)		1.05 (0.21)	1.20 (0.40)	< 0.0001*
Catheter time, mean (SD)		1.05 (0.21)	1.20 (0.21)	< 0.0001*
Use of access sheath and dilation, n (%)	Yes	0 (0)	90 (90)	< 0.0001*
	No	100 (0)	10 (10)	
Post operative fever, n (%)	Yes	5 (5)	20 (20)	0.002*
	No	95 (95)	80 (80)	
Stone free after one month, n (%)	Sucess	96 (96)	93 (93)	0.072
QoL score, mean (SD)		4.30 (0.70)	4.23 (0.73)	0.75

SD: Standard Deviation. QoL: quality of life, * statistically significant

## Discussion

Multiple studies have evaluated the effectiveness of initial decompression strategies, particularly PCN and DJ ureteral stenting, in the management of obstructive calcular pyelonephritis and urosepsis. Although both techniques are widely used, the decision often depends on institutional protocols, anatomical factors, and surgeon experience. PCN is generally considered to provide rapid and reliable drainage in critically ill patients, whereas DJ stenting offers a more straightforward pathway to subsequent RIRS [16, 17]. Clinically, preoperative DJ placement typically facilitates RIRS, while PCN drainage is more frequently followed by PCNL [7]. However, much of the current evidence is derived from retrospective studies, and high-quality prospective data addressing this question remain limited [10].

To the best of our knowledge, this is the first prospective study directly comparing the influence of initial drainage by PCN versus DJ stenting on subsequent RIRS outcomes. Our results question the traditional treatment paradigm in which PCN drainage usually directs management toward PCNL, while DJ placement is followed by RIRS. The present data indicate that both decompression techniques provide equally feasible access to successful RIRS, supporting a more individualized and flexible approach to surgical planning.

In this cohort, demographic characteristics and baseline clinical variables were comparable between the PCN and DJ groups. A noteworthy observation was the high proportion of diabetic patients, consistent with previous reports showing that diabetes increases the likelihood of positive stone cultures even when urine cultures are negative [18]. No significant differences were detected between groups with respect to hydronephrosis severity. Furthermore, the even distribution of stone locations across groups is clinically relevant, as anatomical site has been shown to influence the success of urinary drainage [19].

Our data indicate that PCN was more effective than DJ stenting in achieving rapid resolution of sepsis-related parameters. Patients treated with PCN experienced earlier normalization of body temperature and leukocyte counts compared with those managed with DJ drainage. These results are consistent with the findings of Xu et al. [6], who recommended PCN as the preferred emergency drainage method for patients with urosepsis, particularly in cases of high fever and severe systemic inflammation. In contrast, earlier randomized trials reported no significant difference between PCN and DJ stenting with regard to fever resolution time [9].

The benefits of PCN extended beyond the acute phase and influenced subsequent RIRS outcomes. Specifically, the lower incidence of postoperative fever in the PCN group supports the assumption that maintaining low intrarenal pressure during drainage creates a more favorable environment for endourological interventions [10]. This observation is especially important, as postoperative infections are often linked to bacterial persistence within stone fragments [20]. Because many antibiotics fail to adequately penetrate the stone matrix [21], effective drainage plays a more critical role than antimicrobial therapy alone. This is further underscored by the frequent detection of pathogens in stone cultures that are absent from preoperative urine cultures [22].

The physiological benefits of PCN have been highlighted in several mechanistic studies, demonstrating its ability to reduce intrapelvic pressure during RIRS irrigation [8]. In the present study, PCN was performed by urologists under ultrasonographic guidance, which allowed the procedure to be safely carried out at the bedside, without fluoroscopy, and under local anesthesia. These practical advantages contribute to our conclusion that PCN offers superiority over DJ stenting, a finding consistent with earlier reports [23, 24].

It should be noted, however, that some randomized trials reported no significant differences in outcomes between PCN and DJ stenting [25]. This discrepancy may be explained by differences in study populations, as those trials frequently included patients without infection, whereas our cohort exclusively comprised individuals presenting with sepsis. The reduction in postoperative fever observed in our study is in agreement with large database analyses showing that early urinary decompression lowers sepsis-related mortality [26]. Conversely, our results differ from other randomized trials that found no advantage of one drainage method over the other in terms of infection resolution [14]; this may be attributable to our stricter inclusion criteria and focus on septic patients only.

Our findings indicate that PCN may provide advantages over DJ stenting with respect to operative efficiency, recovery, and postoperative complications. Operative time was shorter in the PCN group, likely because there was no need for prior stent removal or access sheath placement, and intraoperative visualization was clearer due to continuous drainage despite high irrigation pressures. The shorter operative duration may also explain the reduced incidence of postoperative fever in this group. These observations contrast with previous reports that found no significant difference in operative parameters [10]. Although stone-free rates were slightly higher in the PCN group, the difference did not reach statistical significance, which is consistent with earlier findings [10].

It should be noted that our comparison was between two complete treatment pathways: the PCN pathway followed by RIRS, and the DJ stenting pathway followed by RIRS. In our protocol, the use of a UAS is an integral part of the DJ-RIRS pathway to facilitate scope passage and reduce intrarenal pressure. Conversely, the existing PCN tract serves as a natural access and drainage channel, negating the need for a UAS in the PCN group. Therefore, the observed differences in shorter operative time and lower incidence of postoperative fever in the PCN group may be partially attributable to the avoidance of UAS use, underscoring the superiority of the PCN pathway as a comprehensive treatment strategy in this patient cohort.

With regard to patient-reported outcomes, this study demonstrated no significant differences in quality-of-life (QoL) scores between the two groups. This result differs from previous research, which reported superior QoL in patients treated with PCN compared with those managed with DJ stents [27]. Similarly, de Sousa Morais et al. [28] observed a decline in QoL before and after intervention in DJ stent patients but not in those managed with PCN. Mokhmalji et al. [25] also concluded that DJ stenting may negatively affect QoL relative to PCN. Furthermore, the shorter hospital stay associated with PCN in our study supports prior evidence that PCN may offer favorable effects on overall QoL [29].

Our study has several limitations. Being a single-center trial may restrict the external validity of the results. The absence of long-term follow-up also limits the evaluation of late complications and stone recurrence. In addition, intrarenal pressure was not measured, although this parameter is considered critical for understanding postoperative infection risk [10]. All patients in the DJ group underwent the procedure with the use of a ureteral access sheath. As a result, subgroup analysis comparing DJ patients with and without access sheath use is not feasible. Another limitation is the use of a general quality-of-life scale rather than a disease-specific tool, which may have underestimated differences between the groups. Despite these shortcomings, the prospective randomized design and relatively large sample size enhance the robustness of the findings.

## Conclusion

Both DJ stenting and PCN are effective decompression methods that allow successful subsequent RIRS. However, in patients with obstructive pyelonephritis, PCN appears to provide superior infection control, faster clinical recovery, shorter operative times, and fewer postoperative fevers, suggesting an advantage over DJ stenting in septic cases.

## Data Availability

The datasets used and/or analyzed during the current study are available from the corresponding author on reasonable request.

## Abbreviations

CBC: Complete Blood Count
CT: Computed Tomography
DJ: Double-J stent
PCN: Percutaneous Nephrostomy
PCNL: Percutaneous Nephrolithotomy
RIRS: Retrograde Intrarenal Surgery
SD: Standard Deviation
WBC: White Blood Cells
WHO: World Health Organization
QOL: World Health Organization Quality of Life (short form)
qSOFA: Quick Sequential Organ Failure Assessment
